# STAT3基因突变的T细胞大颗粒淋巴细胞白血病临床特征研究

**DOI:** 10.3760/cma.j.issn.0253-2727.2021.06.009

**Published:** 2021-06

**Authors:** 海玲 刘, 蕾 曹, 晓丽 赵, 晶晶 郭, 纯 乔, 华渊 朱, 莉 王, 卫 徐, 建勇 李, 磊 范

**Affiliations:** 1 南京医科大学第一附属医院、江苏省人民医院血液科 210029 Department of Hematology, Jiangsu Province Hospital, The First Affiliated Hospital of Nanjing Medical University, Nanjing 210029, China; 2 南京市第二人民医院血液科 210029 Department of Hematology, Nanjing Second Hospital, Nanjing 210029, China

**Keywords:** 白血病，大颗粒淋巴细胞, 基因，STAT3, 免疫抑制, 治疗, Leukemia, large granular lymphocyte, Gene, STAT3, Immunosuppression, Therapy

## Abstract

**目的:**

探讨携带STAT3基因突变的T细胞大颗粒淋巴细胞白血病（T-LGLL）患者的临床特征，为此类患者的临床管理提供参考。

**方法:**

回顾性分析2009至2019年就诊于江苏省人民医院的T-LGLL患者的临床资料，比较STAT3突变患者与未突变患者的基线临床数据、治疗反应及生存结局。

**结果:**

共纳入80例患者，STAT3未突变组66例，STAT3突变组14例（17.5％），其中Y640F突变发生频率最高（42.9％）。STAT3突变组与STAT3未突变组相比，HGB减低（67.5 g/L对82.5 g/L，*P*＝0.018），中性粒细胞计数减少（0.665×10^9^/L对1.465×10^9^/L，*P*<0.001），乳酸脱氢酶升高（229 U/L对198 U/L，*P*＝0.041），铁蛋白升高（402.5 g/L对236.0 g/L，*P*＝0.029），TCR Vβ亚家族表达率升高（89.2％对65.4％，*P*＝0.014），具备治疗指征患者比例升高（100％对74％，*P*＝0.033）。STAT3突变组与未突变组一线免疫抑制治疗的完全缓解率分别为38.5％和32.7％，差异无统计学意义（*P*＝0.748）。STAT3突变组与未突变组一线免疫抑制治疗的总有效率分别为69.2％和69.4％，差异无统计学意义（*P*＝1.000）。中位随访63（2～121）个月，两组总生存时间（均未达到）的差异无统计学意义（*P*＝0.170）。

**结论:**

STAT3基因突变的T-LGLL患者可能有更高的肿瘤负荷和治疗需求，一线应用免疫抑制剂疗效良好。STAT3基因突变对T-LGLL患者预后的意义尚需进一步验证。

## Abstract (en)

**Objective:**

This study aims to explore the clinical characteristics of T-cell large granular lymphocyte leukemia（T-LGLL）patients with STAT3 mutation status and provide a reference for clinical management of such patients.

**Methods:**

The clinical data of T-LGLL patients between 2009 and 2019 in Jiangsu Province Hospital were retrospectively analyzed. Differences in baseline clinical data, treatment responses, and survival outcomes in patients with STAT3 mutations or with no mutations were compared.

**Results:**

A total of 80 patients were included, including 66 patients without STAT3 mutation and 14 patients（17.5％）with STAT3 mutation. The frequency of Y640F mutation was the highest（42.9％）. Compared with non STAT3 mutation group, STAT3 mutation group had lower HGB（67.5 g/L *vs* 82.5 g/L, *P*＝0.018）, lower neutrophil count（0.665×10^9^/L *vs* 1.465×10^9^/L, *P*<0.001）, higher LDH（229 U/L *vs* 198 U/L, *P*＝0.041）, higher ferritin（402.5 g/L *vs* 236.0 g/L, *P*＝0.029）, higher expression rate of TCR Vβ subfamily（89.2％ *vs* 65.4％, *P*＝0.014）and higher proportion of patients with treatment indications（100％ *vs* 74％, *P*＝0.033）. The complete remission rates of STAT3 mutation group and non mutation group were 38.5％ and 32.7％, respectively, with no significant difference （*P*＝0.748）. The overall response rate of first-line immunosuppressive therapy in STAT3 mutation group and non mutation group were 69.2％ and 69.4％, respectively, with no significant difference（*P*＝1.000）. The median follow-up time was 63（2-121）months. There was no significant difference in the overall survival time between the two groups（*P*＝0.170）.

**Conclusion:**

T-LGLL patients with STAT3 mutations seems to be correlated with an increased tumor burden and high treatment demand, and had a good response to first-line immunotherapies. The prognostic significance of STAT3 mutation in T-LGLL patients requires further validation.

大颗粒淋巴细胞白血病（LGLL）是一种罕见的克隆性淋巴组织增殖性疾病，发病率约为0.2/10 000 000人年[1]。2008年WHO根据精确的细胞起源和临床特征将该病分为T细胞LGLL（T-LGLL）、NK细胞慢性淋巴增殖性疾病和侵袭性NK细胞白血病三种亚型[2]，其中T-LGLL最常见，约占所有病例的85％[3]，但其发病机制尚未明确，一般认为是由抗原长期刺激免疫系统导致细胞毒性T细胞由多克隆增殖逐步转变为寡克隆及单克隆增殖所致，其中涉及多种信号通路的失调，包括Fas-FasL、Ras-MEK1-ERK、PI3K-Akt、IL-15、JAK-STAT、NF-κB等[4]–[5]。这些通路中，JAK-STAT通路的异常激活在T-LGLL的发生发展中起重要作用[6]，信号转导和转录激活蛋白（signal transducer and activator of transcription，STAT）3基因的点突变是最常见的遗传学异常[7]。JAK或其他类型酪氨酸激酶异常激活会导致STAT（尤其是STAT3）持续激活，这种蛋白已在超过20种肿瘤细胞中被发现，与细胞增殖、存活和恶性转化相关[8]。但与其他癌症相比，我们对T-LGLL中JAK-STAT通路的认知仍然非常有限[9]。2001年，在LGLL中首次发现了STAT3的组成性激活[10]。2012年，Koskela等[11]报道40％（31/77）的LGLL患者存在STAT3的Src同源区2（Src homology 2，SH2）结构域体细胞突变，该区域是介导STAT3蛋白二聚化和激活的关键区域。2016年WHO分型将2012年新发现的STAT3基因突变纳入分类中[12]–[13]。鉴于STAT3基因在LGLL致病中的重要地位，对其进行进一步研究非常必要。因此，本文主要对本中心T-LGLL患者进行回顾性分析，以提高对携带STAT3基因突变的T-LGLL患者的认识，加强对此类患者的临床管理。

## 病例与方法

1. 病例：收集2009年8月至2019年9月就诊于江苏省人民医院的120例成人T-LGLL患者的临床资料，包括症状、体征、实验室检查、治疗情况等。80例患者接受了STAT3基因检测，14例（17.5％）突变阳性，11例行STAT5b基因检测，结果均为阴性。将80例有STAT3基因检测结果的患者纳入本研究，所有入组成员满足如下诊断标准[14]：①外周血大颗粒淋巴细胞（LGL）计数>0.5×10^9^/L且持续超过6个月；②具有典型的免疫表型，共表达CD3^+^CD4^−^CD8^+^CD57^+^TCRαβ^+^，少数患者为变异亚型，包括CD4^+^CD8^−^TCRαβ^+^、CD4^+^ CD8^+^ TCRαβ^+^或CD4^−^CD8^−^TCRγδ^+^等；③用PCR和Sanger法检测TCR基因重排，或用流式细胞术检测到TCR Vβ区的限制性；④临床表现包括血细胞减少、脾大、纯红细胞再生障碍（PRCA）和类风湿关节炎（RA）等。前3条标准必须满足，但对于25％～30％ LGL计数<0.5×10^9^/L的患者，若有临床症状或克隆性证据也可诊断为T-LGLL。治疗指征：与中性粒细胞减少相关的反复感染、中性粒细胞绝对计数<0.5×10^9^/L、症状性或输血依赖性贫血、PLT<50×10^9^/L、有全身症状或脾肿大、伴需要治疗的自身免疫性疾病[14]。本研究符合赫尔辛基宣言，且获得江苏省人民医院医学伦理委员会的批准。

2. STAT3基因突变检测：①DNA提取：所有患者均在获得知情同意后抽取新鲜骨髓标本。按QIAamp^®^Blood DNA Mini Kit试剂盒说明书提取基因组DNA，用Eppendorf分光光度计检测提取的单个核DNA浓度和纯度，置于−20 °C保存备用。②引物设计：从GENEBANK上查找STAT3基因序列（NC-000017.11），应用PRI MER EXPRESS软件设计相应的引物。外显子20正义链：5′-CAAGGTGTCCTCTACAAAGATAAAG-3′；反义链：5′-CCACTGTGTTAGACATAAAGAAGAC-3′；外显子21正义链：5′-AAAAGACAAAATTCTTGGCACCTCC-3′；反义链：5′-GAATAATCTGGCATATCCCTGTGG-3′。③PCR扩增：PCR总体系20 µl，含2×Taq PCR Master Mix 10 µl、正反义引物各1 µl、cDNA模板2 µl、双蒸水6 µl。循环条件：94 °C预变性4 min，94 °C变性30 s，58 °C退火45 s，72 °C延伸1 min，共35个循环。72 °C后延伸10 min，4 °C保存。④测序：PCR产物使用一代Sanger法测序，结果与正常STAT基因序列比对。

3. 治疗方案、疗效判定、生存评估及随访[14]–[15]：由于临床证据有限，T-LGLL的治疗尚无标准方案。临床常用的口服药物包括下列3种：①甲氨蝶呤（MTX），每周10 mg/m^2^；②环磷酰胺（CTX），每日50～100 mg；③环孢素A（CsA），5～10 mg·kg^−1^·d^−1^，分两次口服。激素单用效果不佳，多作为辅助用药。选用其中一种方案，治疗4个月后评估疗效。血液学完全缓解（CR）定义为外周血细胞及LGL计数正常；血液学部分缓解（PR）指外周血细胞计数改善（中性粒细胞绝对计数增加>50％，HGB增加>10 g/L或每月输血量减少>50％，至少持续4个月）但未达CR标准；治疗失败指治疗4个月未达到缓解或缓解后再次恶化。研究的主要终点包括总反应率（ORR）和CR率（CRR），前者指达到PR和CR患者的总比例；次要终点为总生存（OS）期，即从疾病确诊至死亡或随访结束时间。随访截止时间为2020年2月。

4. 统计学处理：应用Stata MP 13.1软件进行分析。分类变量组间比较采用*χ*^2^检验或Fisher精确检验，连续变量用中位数（范围）描述，组间比较采用非参数秩和检验（Mann-Whitney *U*检验）。利用Kaplan-Meier法绘制生存曲线。*P*<0.05为差异有统计学意义。

## 结果

1. 临床特征：在80例患者中，14例（17.5％）患者STAT3基因突变阳性，其中5例（35.7％）外显子20突变阳性，包括E616V（1/14，7.1％）、S614R（1/14，7.1％）、SNPrs117691970（3/14，21.4％）；9例（64.3％）外显子21突变阳性，包括Y640F（6/14，42.9％）、V671F（1/14，7.1％）、N647I（1/14，7.1％），1例类型不详（1/14，7.1％）。突变组与未突变组的临床特征比较见表1。结果显示，STAT3突变与HGB减低（*P*＝0.018）、中性粒细胞减少（*P*<0.001）、LDH升高（*P*＝0.041）、铁蛋白（FER）升高（*P*＝0.029）及TCR Vβ亚家族表达率高相关（*P*＝0.014）。少数病例（10/60，16.7％）表现为染色体异常，然而未发现特异性结构和（或）数目改变，STAT3基因突变组与未突变组间差异无统计学意义（*P*＝0.671）。

**表1:** STAT3突变与未突变T细胞大颗粒淋巴细胞白血病患者临床特征比较

特征	STAT3基因		*χ*^2^或*z*值	*P*值
	未突变组（66例）	突变组（14例）		
性别（例，男/女）	32/34	10/4	2.438	0.118
年龄［岁，*M*（范围）］	59（31～89）	59（39～76）	0.367	0.713
自身免疫性疾病（例，有/无）	9/57	2/12	−	1.000
RA（例，有/无）	2/64	0/14	−	1.000
PRCA（例，有/无）	13/53	6/8	−	0.086
B症状（例，有/无）	12/54	1/13	−	0.446
肝肿大（例，有/无）	2/64	1/13	−	0.443
脾肿大（例，有/无）	14/52	5/9	−	0.302
淋巴结肿大（例，有/无）	10/56	2/12	−	1.000
骨髓LGL浸润（例，有/无）	54/12	12/2	−	1.000
免疫表型（例，典型/非典型）	21/45	1/13	−	0.097
ECOGPS评分［分，*M*（范围）］	2（0～4）	2（0～2）	0.447	0.655
aCCI［分，*M*（范围）］	2（0～6）	1.5（0～3）	0.704	0.481
LGL计数［×10^9^/L，*M*（范围）］	2.94（0.38～19.75）	2.83（0.63～9.80）	0.249	0.803
WBC［×10^9^/L，*M*（范围）］	6.225（1.60～26.30）	5.615（1.40～12.92）	1.589	0.112
ALC［×10^9^/L，*M*（范围）］	3.76（0.80～23.03）	3.82（0.97～12.10）	0.101	0.919
ANC［×10^9^/L，*M*（范围）］	1.465（0.21～9.18）	0.665（0.05～1.84）	3.685	<0.001
HGB［g/L，*M*（范围）］	82.5（41～159）	67.5（31～108）	2.368	0.018
PLT［×10^9^/L，*M*（范围）］	189（11～625）	204（47～478）	−0.025	0.980
LDH［U/L，*M*（范围）］	198（94～627）	229（180～462）	−2.042	0.041
FER［µg/L，*M*（范围）］	236.0（6.9～2180.0）	402.5（171.0～997.2）	−2.182	0.029
染色体核型（例，异常/正常）	9/40	1/10	−	0.671

注：STAT3：信号转导和转录激活蛋白3；RA：类风湿关节炎；PRCA：纯红细胞再生障碍；B症状：发热、盗汗、体重减轻；LGL：大颗粒淋巴细胞；ECOG PS：美国东部肿瘤协作组体能状态；aCCI：年龄校正Charlson合并症指数；ALC：淋巴细胞绝对计数；ANC：中性粒细胞绝对计数；FER：铁蛋白；−：因该分类变量采用Fisher精确检验，故无*χ*^2^值

在80例患者中，61例患者进行了TCR Vβ亚家族检测，其中12例表达正常，11例患者24种亚单位总表达率减低，38例（62.3％）出现单一亚家族单克隆表达。STAT3突变组与未突变组患者表达类型的差异无统计学意义（*P*>0.05）。38例TCR Vβ亚家族限制性表达患者的中位表达率为68.65％（14.1％～98.0％），STAT3无突变组中位表达率为65.4％（14.1％～97.4％），突变组中位表达率为89.2％（36.2％～98.0％），组间比较差异有统计学意义（*P*＝0.014）。

2. 治疗方案和疗效评价：至随访结束，STAT3突变组14例患者均有治疗指征，STAT3未突变组49例患者有治疗指征，两组的差异有统计学意义（*P*＝0.033）。1例STAT3 Y640F突变者拒绝治疗，其余62例患者接受治疗（表2）。STAT3未突变组一线治疗的ORR为69.4％（34/49），CRR为32.7％（16/49）；28例患者接受MTX±激素作为一线方案，10例达CR，12例达PR；13例患者接受CsA±激素作为一线方案，4例达CR，3例达PR；2例患者接受CTX±激素作为一线方案，均达PR；其余6例接受其他治疗，包括单用激素1例（治疗失败）、CHOP方案（环磷酰胺+多柔比星+长春新碱+泼尼松）1例（治疗失败）、CEOP方案（环磷酰胺+表阿霉素+长春新碱+泼尼松）1例（达PR）、沙利度胺2例（1例达CR，1例治疗失败）、硼替佐米1例（达CR）。STAT3突变组ORR为69.2％（9/13），CRR为38.5％（5/13）。STAT3 Y640F突变患者中4例予MTX治疗（1例达CR，2例达PR，1例治疗失败），1例予CsA治疗（达CR）；非Y640F突变患者中6例予MTX治疗（3例达CR，2例达PR，1例治疗失败），2例予CsA治疗（均治疗失败）。STAT3突变组与无突变组一线治疗后CRR和ORR、一线MTX治疗后CRR和ORR的差异均无统计学意义（表2）；STAT3突变组一线MTX和CsA治疗后CRR和ORR的差异均无统计学意义（*P*值分别为1.000、0.203）；STAT3 Y640F突变患者与非Y640F突变患者一线治疗后CRR和ORR（*P*值分别为1.000、0.203）、一线MTX治疗后CRR和ORR（*P*值分别为0.571、1.000）的差异均无统计学意义。

**表2:** STAT3突变与未突变T细胞大颗粒淋巴细胞白血病患者不同治疗方案下疗效比较

组别	例数	完全缓解率（％）				总缓解率（％）			
		MTX	CsA	CTX	总体	MTX	CsA	CTX	总体
STAT3突变组	13	40.0	33.3	0	38.5	80.0	33.3	0	69.2
STAT3未突变组	49	35.7	30.8	0	32.7	78.6	53.8	100	69.4

*P*值		1.000	1.000	−	0.748	1.000	1.000	−	1.000

注：STAT3：信号转导和转录激活蛋白3；MTX：甲氨蝶呤；CsA：环孢素A；CTX：环磷酰胺；−：无数据。STAT3突变组应用MTX、CsA、CTX治疗者分别为10、3、0例，STAT3未突变组分别为28、13、2例

3. STAT3基因突变对预后的影响：中位随访63（2～121）个月，STAT3突变组的10年OS率为100％，中位生存时间未达到。STAT3未突变组的10年OS率为82.1％，中位生存时间未达到。STAT3突变组与未突变组OS的差异无统计学意义（*P*＝0.170）（图1）。

**图1:**
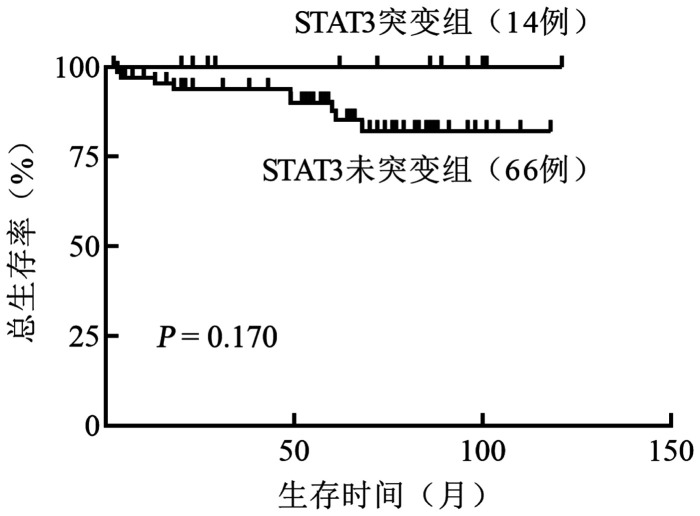
STAT3突变和未突变组T细胞大颗粒淋巴细胞白血病患者的总生存曲线

## 讨论

STAT3基因是STAT家族的重要成员之一，也是首个LGLL高度特异的分子标记。在T-LGLL患者中，STAT3基因的突变热点位于20、21外显子，主要分布于SH2功能区。STAT3基因发生突变激活后，SH2区与受体中被磷酸化的酪氨酸残基结合，同时自身发生磷酸化形成二聚体，穿过细胞核膜移至细胞核内，成为增强抗细胞凋亡途径的转录因子，引起细胞的异常增殖和恶性转化[8]。目前，国外已有学者对STAT3基因突变与T-LGLL患者临床特征间的相关性进行了报道，但中国人群的相关研究仍缺乏。

既往研究显示，T-LGLL患者的STAT3突变阳性率有较大差异（11.1％～75.0％）[7],[11],[16]–[26]，其原因可能包括：①入组病例的差异。Jerez等[26]的研究认为STAT3突变检出率与LGL克隆大小相关，大克隆的LGL扩增中STAT3突变更常见，而一部分患者有多种小克隆LGL扩增，突变检出率相对较低。②检测技术的差异。Sanger测序技术目前最常用，要求患者至少有10％～20％的突变克隆细胞，阳性率为11.1％～72.7％[23]–[24]。为进一步提高检出率，有学者将Sanger测序和等位基因特异性PCR（AS-PCR）结合，阳性率为37.6％～47.6％[18],[21]。Rajala等[20]还将Sanger测序和扩增子测序进行比较，发现后者具有更高的灵敏度（23％对38％）。迄今为止，阳性率最高（75.0％）的检测方法是二代测序技术[22]。在样本量相似情况下，使用二代测序技术或联合应用Sanger测序和AS-PCR可能获得更高的阳性率。③样本量的差异。由于该病罕见，临床数据收集困难，约80％的研究不超过百例，样本量过小会带来不可避免的抽样误差。在我们的研究中，17.5％（14/80）的患者检测出STAT3突变，低于上述大部分研究，考虑原因可能与检测样本量较小、LGL克隆水平较低和检测技术敏感性较差有关，进一步扩大样本、建立LGL细胞分选流程及提高检测技术的敏感性可能会提高阳性率。我们通过外周血LGL绝对计数和TCR Vβ亚家族的表达率评估该疾病的克隆大小。虽然STAT3突变状态与LGL计数间未显示相关性，但突变患者有更高的TCR Vβ亚家族单克隆表达率，这一结论与Jerez等[26]的报道相似。

2012年有研究报道STAT3突变与症状性疾病之间存在显著相关性，突变者较未突变者更易出现中性粒细胞减少、RA和自身免疫性溶血性贫血，PRCA病例仅发现于无突变患者[26]。2013年Qiu等[25]的报道纳入28例中国T-LGLL患者，STAT3突变与β_2_-微球蛋白水平升高、中性粒细胞计数减少、PRCA相关。RA、PRCA与STAT3突变状态的关系尚存在争议[20],[25]–[27]，可能与其在T-LGLL患者中的发生率不同有关。西方国家T-LGLL患者的伴随疾病以RA最为常见，我国的报道则以PRCA多见[14],[28]。日本的一项研究在LGLL相关性PRCA患者中发现77％有STAT3突变，表明突变克隆可能直接参与了对红细胞生成的破坏[20]。本研究中，PRCA病例占23.8％，而仅观察到2.5％的RA病例，后者的比例明显低于国外报道[29]。突变组中PRCA的发生率具有更高的趋势，RA仅存在于未突变组。与既往文献一致，中性粒细胞和HGB减低与STAT3突变相关[16],[30]。Mariotti等[31]的研究认为病理性LGL骨髓浸润在中性粒细胞减少症的发病机制中仅发挥很小的作用，并提出STAT-miR146b-FasL轴参与该过程，一旦触发该信号，会导致Fas配体大量产生，从而介导中性粒细胞凋亡。贫血的发病机制包括高发病率的PRCA和自身免疫性溶血性贫血，但STAT3在其中发挥的作用尚不清楚。此外，Shi等[17]的研究认为T-LGLL患者在STAT3突变状态下具有更高的肿瘤负荷，在本研究中我们发现STAT3突变与较高的LDH、FER及TCR Vβ表达率有关，与外周血LGL绝对计数无明显相关性，虽然LDH和FER在多种血液系统疾病中可反映一定程度的肿瘤负荷，TCR Vβ表达率也可评估疾病克隆大小，但由于缺乏流式细胞术等支持证据，我们选择更加保守地推测该结论。

T-LGLL属于惰性T淋巴细胞增殖性肿瘤，临床多进展缓慢，对于无指征者可随访观察，但大多数患者最终需要治疗。多项研究均提及基线状态下携带STAT3突变的患者具有更高的治疗需求[16],[32]，我们的研究结果与其一致，STAT3突变患者均具有治疗指征。由于该病较为罕见，临床治疗策略多基于回顾性研究的结果，目前免疫抑制剂处于一线地位[33]。2015年Loughran等[15]研究了STAT3基因与免疫抑制剂疗效的潜在相关性，结果显示，突变患者对MTX治疗反应更好，尤其是STAT3 Y640F突变基因型患者。Shi等[17]也报道MTX对STAT3突变患者显示出良好的疗效。上述结论均表明STAT3突变很可能为T-LGLL患者一线治疗方案的选择提供参考。本研究中，STAT3 Y640F突变基因型患者最多见，约占所有突变患者的42.9％，其对一线MTX的治疗反应率达到了75％（3/4），虽然与其他突变基因型患者相比差异无统计学意义，但该结果可能受到了样本量的限制。既往研究认为STAT3激活与耐药性表型相关[34]–[35]，在T-LGLL患者中似乎有不同的结果。STAT3突变状态在T-LGLL患者中的预后意义仍有争议，Sanikommu等[32]认为突变患者总体生存更好，Barilà等[16]的研究显示STAT3突变对患者的生存具有不利影响。本研究未观察到STAT3突变对患者生存的显著影响，可能与随访时间和样本量有限相关。截至随访结束，突变组中未观察到患者死亡，两组患者OS的差异无统计学意义，有研究报道类似结论[23],[26]。未来需要延长随访时间或扩大样本量进一步探讨STAT3突变在T-LGLL中的预后价值。

总而言之，携带STAT3基因突变的成人T-LGLL患者似乎有更高的肿瘤负荷和更早的治疗需求，其对免疫抑制剂尤其是MTX治疗反应良好。本研究尚未发现STAT3基因突变对T-LGLL预后的意义，但其有望成为T-LGLL患者治疗指征和方案选择的参考因素，值得进一步探索。
